# Adipose tissue–derived stromal cells’ conditioned medium modulates endothelial‐mesenchymal transition induced by IL‐1β/TGF‐β2 but does not restore endothelial function

**DOI:** 10.1111/cpr.12629

**Published:** 2019-08-29

**Authors:** Tácia Tavares Aquinas Liguori, Gabriel Romero Liguori, Luiz Felipe Pinho Moreira, Martin Conrad Harmsen

**Affiliations:** ^1^ Laboratório de Cirurgia Cardiovascular e Fisiopatologia da Circulação (LIM‐11), Faculdade de Medicina Instituto do Coração (InCor), Hospital das Clinicas HCFMUSP, Universidade de Sao Paulo Sao Paulo Brazil; ^2^ Department of Pathology and Medical Biology University of Groningen, University Medical Center Groningen Groningen The Netherlands

**Keywords:** adipose tissue–derived stromal cells, endothelial‐mesenchymal transition, human umbilical vein endothelial cells, TGF‐β

## Abstract

**Objectives:**

Endothelial cells undergo TGF‐β–driven endothelial‐mesenchymal transition (EndMT), representing up to 25% of cardiac myofibroblasts in ischaemic hearts. Previous research showed that conditioned medium of adipose tissue–derived stromal cells (ASC‐CMed) blocks the activation of fibroblasts into fibrotic myofibroblasts. We tested the hypothesis that ASC‐CMed abrogates EndMT and prevents the formation of adverse myofibroblasts.

**Materials and methods:**

Human umbilical vein endothelial cells (HUVEC) were treated with IL‐1β and TGF‐β2 to induce EndMT, and the influence of ASC‐CMed was assessed. As controls, non‐treated HUVEC or HUVEC treated only with IL‐1β in the absence or presence of ASC‐CMed were used. Gene expression of inflammatory, endothelial, mesenchymal and extracellular matrix markers, transcription factors and cell receptors was analysed by RT‐qPCR. The protein expression of endothelial and mesenchymal markers was evaluated by immunofluorescence microscopy and immunoblotting. Endothelial cell function was measured by sprouting assay.

**Results:**

IL‐1β/TGF‐β2 treatment induced EndMT, as evidenced by the change in HUVEC morphology and an increase in mesenchymal markers. ASC‐CMed blocked the EndMT‐related fibrotic processes, as observed by reduced expression of mesenchymal markers *TAGLN* (*P* = 0.0008) and *CNN1* (*P* = 0.0573), as well as SM22α (*P* = 0.0501). The angiogenesis potential was impaired in HUVEC undergoing EndMT and could not be restored by ASC‐CMed.

**Conclusions:**

We demonstrated that ASC‐CMed reduces IL‐1β/TGF‐β2‐induced EndMT as observed by the loss of mesenchymal markers. The present study supports the anti‐fibrotic effects of ASC‐CMed through the modulation of the EndMT process.

## 1 INTRODUCTION

Heart failure (HF) is an irreversible and potentially lethal clinical condition that affects nearly 23 million people worldwide.^1^ The five‐year survival is approximately 50%. Obviously, HF impacts significantly on the quality of life and is an increasing burden on society and health care. Heart failure presents as various forms of idiopathic or heritable cardiomyopathy and as the consequence of adverse cardiac tissue remodelling after acute myocardial infarction.

In normal physiology, cardiac tissue is in homeostasis that is maintained by a well‐regulated biochemical and biomechanical crosstalk between the parenchyma and the supportive tissue stroma. Cardiac parenchyma comprises cardiomyocytes, while the stroma consists of vasculature, fibroblasts and their product, the extracellular matrix (ECM). The ECM provides structural support and architecture and instructs adhered tissue cells.^2^ HF disrupts the cardiac tissue homeostasis. A prominent feature is the proliferation of myofibroblasts and their excessive deposition and accumulation of fibrotic ECM. Thus, HF is a process of cardiac fibrosis. Differentiation of cardiac fibroblasts to myofibroblasts is a major contribution to HF.^3^, ^4^ Several other cell types, both endogenous in the heart and exogenous, also contribute to cardiac fibrosis.^5^ The heart is particularly rich in capillaries and thus endothelial cells. Under pathological conditions, endothelial cells contribute to adverse wound healing and tissue remodelling via endothelial‐mesenchymal transition (EndMT) and contribute significantly to cardiac fibrosis and development of heart failure.^6^, ^7^ After acute myocardial infarction in mice, up to 25% of cardiac myofibroblasts are the consequence of EndMT.^8^ Irrespective of the source, *for example,* fibroblasts or endothelial cells, the resulting myofibroblasts are indistinguishable with respect to proliferation and ECM remodelling. Interestingly, EndMT is pivotal during cardiogenesis, when EndMT underlies the development of heart valves.^9^ In contrast, in adult life, EndMT is related to pathophysiological phenomena such as cardiac fibrosis,^10^, ^11^, ^12^ after myocardial infarction,^8^, ^13^ diabetic cardiomyopathy^14^, ^15^ and hypertensive heart disease.^11^, ^16^ Therefore, inhibition or reversal of cardiac EndMT is a therapeutic option to interfere with heart failure.

Endothelial‐to‐mesenchymal transition is a relatively slow dedifferentiation process (days to weeks) that is driven by pro‐fibrotic growth factors of the TGF‐ß superfamily,^17^, ^18^ such as TGF‐β2. Several processes coincide: endothelial cells loose cell‐to‐cell contacts and the downregulated endothelial markers. This causes the cells to transit from their characteristic cobblestone morphology to a spindle‐like shape. Simultaneously, a progressive upregulation of mesenchymal markers occurs, such as smooth muscle protein 22 alpha (SM22α), calponin and alpha‐smooth muscle actin (αSMA). Similar to myofibroblasts, the EndMT process renders cells highly migratory and proliferative, while these become resistant to apoptosis too. The process of EndMT also coincides with the increased production and deposition of extracellular matrix, which contributes to the development and progression of cardiac fibrosis including the increased stiffness of the failing heart.^19^, ^20^

The TGF‐β superfamily is important during embryogenesis, but also for wound healing, and thus influences cell growth, proliferation, differentiation and migration.^21^ In addition, TGF‐β members are strong regulators of ECM remodelling, in particular, through upregulation of constructive proteins such as collagens. All three TGF‐β isoforms stimulate EndMT.^22^, ^23^, ^24^, ^25^, ^26^, ^27^, ^28^ In cardiovascular wound healing, fibrosis coincides with inflammation. In fact, EndMT is synergized by TGF‐β2 and IL‐1β.^29^, ^30^ Heart failure is also associated with pro‐fibrotic stimuli by members of the TGF‐β superfamily, inflammation and reactive oxygen species (ROS). These three triggers are tightly interrelated because TGF‐ß promotes inflammatory activation via TAK1, similar to ROS, and with it, EndMT.^31^ We have shown that pro‐fibrotic stimuli and pro‐inflammatory stimuli synergize EndMT,^29^, ^30^ and other studies showed that ROS mediates the EndMT process through the TGF‐β superfamily.^32^, ^33^

Cardiac stem cell therapy with mesenchymal stem/stromal cells (MSC) has shown to improve remodelling after acute myocardial infarction. This suggests that MSC affect myofibroblast formation and function. The intramyocardial administration of mesenchymal stromal cells (MSC), which include adipose tissue–derived stromal cells (ASC), has benefit for cardiac function and remodelling in a variety of cardiac diseases.^34^, ^35^, ^36^, ^37^, ^38^, ^39^, ^40^, ^41^, ^42^ As a matter of fact, injection of conditioned medium of MSC (CMed) also improved cardiac function.^43^, ^44^ Previous research in our laboratory showed that ASC secrete paracrine factors that abrogate TGF‐β–induced differentiation of dermal fibroblasts to myofibroblasts which is a mesenchymal transition too.^45^ In general, ASC and their secreted bioactive factors harbour pro‐regenerative 46, 47, 48 and anti‐inflammatory potential.^49^, ^50^ In addition, ASC promote angiogenesis. Therefore, we hypothesized that the formation of myofibroblasts from endothelial cells via EndMT, which also is a TGF‐β–driven process and synergized by IL‐1β, is down‐modulated by the paracrine action of ASC while it would rescue their endothelial phenotype. We tested our hypothesis in vitro, by assessing the influence of adipose tissue–derived stromal cells’ conditioned medium (ASC‐CMed) on pro‐fibrotic and pro‐inflammatory–induced EndMT.

## 2 EXPERIMENTAL

### 2.1 Cell sources, cell culture, conditioned medium and experimental groups

Human umbilical vein endothelial cells (HUVEC) were obtained from the endothelial cell culture facility of our institution and comprised pools of at least three donors. Cells were seeded on gelatin‐coated plates (1% gelatin solution in PBS) at a density of 35 000 cells/cm^2^ and cultured until confluency in endothelial cell medium (ECMed) composed of RPMI 1640 basal medium (#BE04‐558F, Lonza) with 10% heat‐inactivated foetal bovine serum (FBS; #F0804, Sigma‐Aldrich), 1% penicillin/streptomycin (#15140122, Gibco Invitrogen), 1% L‐glutamine (#17‐605E, Lonza BioWhittaker), 5 U/mL heparin (LEO Laboratories Limited) and 50 µg/mL bovine brain extract (BBE, homemade preparation). HUVEC between passages 3 and 6 were used for the experiments. Confluent HUVEC were divided into six groups with different induction/ASC‐CMed combinations, as described in Table 1, and cultured for 5 days. Human recombinant interleukin‐1β (IL‐1β; #200‐01B, PeproTech) and human transforming growth factor beta 2 (TGF‐β2; #100‐35B, PeproTech) were used to stimulate the EndMT process at a concentration of 10ng/mL in all experiments.

**Table 1:** Experimental groups

Group	Description
ECMed	HUVEC culture only with endothelial cell medium
ECMed/IL‐1β	HUVEC culture with endothelial cell medium added with IL‐1β
ECMed/IL‐1β/TGF‐β2	HUVEC culture with endothelial cell medium added with IL‐1β and TGF‐β2
ASC‐CMed	HUVEC culture only with ASC conditioned media
ASC‐CMed/IL‐1β	HUVEC culture with ASC conditioned media added with IL‐1β
ASC‐CMed/IL‐1β/TGF‐β2	HUVEC culture with ASC conditioned media added with IL‐1β and TGF‐β2

ASC, adipose tissue‐derived stromal cells; ASC‐CMed, conditioned media from adipose tissue‐derived stromal cells; ECMed, endothelial culture medium; IL‐1β, human recombinant interleukin‐1 beta; TGF‐β2, human transforming growth factor beta 2; UVEC, human umbilical vein endothelial cells.

Human ASC were isolated as described previously.^51^ Briefly, human abdominal fat was obtained by liposuction. Tissue was washed with phosphate‐buffered saline (PBS) and then enzymatically digested with 0.1% collagenase A (#11088793001, Roche Diagnostic, Mannheim, Germany) in PBS with 1% bovine serum albumin (BSA; #A9647, Sigma‐Aldrich). The tissue was shaken constantly at 37°C for 2 hours. After this, the digested tissue was washed in 1% BSA in PBS and filtered using 70 μm cell strainers. The filtered suspension was centrifuged at 600 ***g*** for 10 minutes, and the cell pellet was resuspended in lysis buffer containing ammonium chloride to remove red blood cells, centrifuged again and resuspended in Dulbecco's modified Eagle's medium (DMEM; #12‐604F, Lonza) with 10% foetal bovine serum (FBS; #F0804, Sigma‐Aldrich), 1% penicillin/streptomycin (#15140122, Gibco Invitrogen) and 1% L‐glutamine (#17‐605E, Lonza BioWhittaker). Cells were cultured at 37°C in a humidified incubator with 5% CO_2_. The medium was refreshed every 2 days. Cells were passed at a ratio of 1:3 after reached confluency. The characterization of the cells was routinely performed as previously described by our group and confirmed the required marker pattern and biological behaviour of ASC.^52^

After the second passage, ASC were maintained in ECMed. ASC‐CMed was obtained from confluent cultures of ASC between passages 3 and 6 from 3 different donors. For ASC‐CMed, cells were cultured in ECMed and the conditioned medium was harvested after 48 hours, filtered in 0.22 µm filters and stored at −20°C until use. The expression of fibroblast growth factor 1 (FGF‐1) and vascular endothelial growth factor (VEGF) was determined in the medium collected from ASC. For this purpose, Magnetic Luminex Human Premixed Multi‐Analyte Kit (R&D Systems) was used according to the manufacturer's protocol. DMEM only was used as negative control.

### 2.2 Immunofluorescence, Gene Expression and Immunoblotting

#### 2.2.1 Immunofluorescence

HUVEC were cultured in 96‐well tissue culture plates in ECMed. After 5 days of EndMT induction, cells were fixed at room temperature with 2% paraformaldehyde (PFA) for 30 minutes. Cells were permeabilized with 1% Triton‐X 100 in PBS at room temperature for 15 minutes and blocked with 5% donkey serum in PBS and 1% BSA at room temperature for 15 minutes. Subsequently, cells were incubated with primary antibodies diluted in 5% donkey serum in PBS at room temperature for 2 hours. The following primary antibodies were used: rabbit anti‐SM22α (1:400; #ab14106, Abcam) and mouse anti‐human PECAM‐1 (1:200; #MAB9381, R&D Systems, Oxon). Controls were incubated with 5% donkey serum in PBS instead of primary antibody. Next, cells were washed with 0.05% Tween‐20 in PBS and incubated with secondary antibodies in 5% donkey serum in PBS with 4',6‐diamidino‐2‐phenylindole (DAPI; 1:5000; #D9542‐5MG, Sigma‐Aldrich) and Alexa Fluor® 488 phalloidin (1:400; #A12379, Life Technologies) at room temperature for 1 hour. The following secondary antibodies were used: donkey anti‐rabbit IgG (H + L) Alexa Fluor® 594 (1:400; #A‐21207, Life Technologies) and donkey anti‐mouse IgG (H + L) Alexa Fluor® 594 (1:400; #ab150108, Abcam). Finally, cells were washed three times with PBS and the plates were imaged with EVOS FL System (Thermo Fisher Scientific) using Texas Red (TXR), DAPI and Green Fluorescent Protein (GFP) channels with 20× magnification.

#### 2.2.2 Gene expression analysis

HUVEC were cultured in 75 cm^2^ flasks. After 5 days of induction, total RNA was isolated using TRIzol reagent (#15596018, Invitrogen Corp) according to the manufacturer's protocol. RNA concentration and purity were determined using a NanoDrop Spectrophotometer (Thermo Scientific). Between 300 ng and 5000 ng of total RNA was used for cDNA synthesis, which was performed using RevertAid^TM^ First Strand cDNA Synthesis Kit (Thermo Fisher Scientific) according to the manufacturer's protocol. The cDNA equivalent of 12 ng total RNA was used per single qPCR. PCR was performed using SYBR Green (Bio‐Rad, Hercules) with the ViiA7 Real‐Time PCR System (Applied Biosystems). Each analysis was done in duplicate for each one of the independent experiments. The primers used are listed in Table S1. Data were analysed using ViiA7 software (Applied Biosystems) and normalized with the ∆*C*_t_ method, using the geometrical mean of 18S ribosomal RNA (*18S RNA*) cycle threshold (*C*_T_) values. The fold‐change in gene expression vs the no treatment control group (ECMed) was calculated using the ∆∆*C*_T_ method.

#### 2.2.3 Immunoblotting analysis

HUVEC were cultured in 75 cm^2^ flasks. After 5 days of induction, cells were rinsed with ice‐cold PBS and lysed in 100 µL of ice‐cold lysis buffer (RIPA; #89900, Thermo Fisher Scientific) containing 1% protease inhibitor cocktail (PIC; #P8340, Sigma‐Aldrich) and 1% Halt™ Phosphatase Inhibitor Cocktail (#78420, Thermo Fisher Scientific). The lysed cells were collected in 2 mL microcentrifuge tubes, and the contents were homogenized by sonication at 30 W for 30 seconds and centrifuged at 7500 ***g*** at 4°C for 5 minutes. The supernatant was collected for the protein concentration determination using the Bio‐Rad DC Protein Assay (#5000112; Bio‐Rad, Hercules) according to the manufacturer's protocol. Gels (12%) were loaded with 25‐30 μg of protein per lane. After electrophoresis, gels were blotted onto nitrocellulose membranes (#170‐4270; Bio‐Rad, Hercules). Blots were blocked with Odyssey® Blocking Buffer (#927‐40000, LI‐COR, Lincoln) in a dilution of 1:1 with PBS at 4°C overnight. Afterwards, blots were incubated with the primary antibodies overnight. The following primary antibodies were used: rabbit anti‐SM22α (1:1000; #ab14106, Abcam), rabbit anti–VE‐cadherin (1:500; #2500S, Cell Signalling), and mouse anti‐GAPDH (1:1000; #ab9484, Abcam). Then, the membranes were washed with Tris‐buffered saline (TBS) with 0.1% Tween‐20 (TBST) 30 minutes and incubated with the Odyssey® secondary antibodies goat anti‐rabbit IRDye 680LT (1:10000; #926‐68021, LI‐COR, Lincoln) and goat anti‐mouse IRDye 800CW (1:10,000; #926‐32210, LI‐COR, Lincoln) for 1 hour. Non‐bound secondary antibodies were removed by washing with TBST for 30 minutes. Then, blots were washed with TBS for 5 minutes and scanned with Odyssey® Infrared Imaging System (LI‐COR, Lincoln).

#### 2.2.4 Endothelial sprouting assay

HUVEC were cultured in 25 cm^2^ flasks. After 5 days of induction, cells were detached from the flasks and counted, and, for each group, 15 000 cells were resuspended in 50 μL of ECMed. Subsequently, cells were seeded in wells of a µ‐Slide Angiogenesis Plate (Ibidi GmbH) previously coated and incubated at 37°C with 10 μL of Matrigel® (#356231, BD Biosciences) for 2 hours. The sprouting was allowed to proceed for 8 hours. Every condition was done in duplicate, and the experiment was performed three times independently. Formation of sprouting networks was imaged with a DM2000 LED Inverted Microscope (Leica) using 2.5× magnification and analysed using ImageJ software. The number of nodes, branches, segments, total length, number of meshes and mean mesh size were analysed.

### 2.3 Statistical analysis

All data were obtained from at least three independent experiments performed in duplicate. Data are presented as the mean ± SE of the mean (SEM). Graphs and statistical analysis were done using GraphPad Prism (version 6.01; GraphPad Software, Inc). Differences among multiple groups were analysed by one‐way ANOVA with Sidak's multiple comparison test for the two groups of interested in each scenario.

## 3 RESULTS

### 3.1 ASC secrete fibroblast growth factor 1 (FGF‐1) and vascular endothelial growth factor (VEGF)

**Figure 1:**
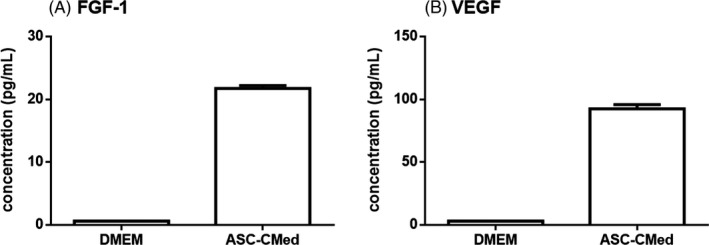
Concentration of growth factors released by ASC in the conditioned medium. A, FGF‐1 B, VEGF

The growth factor release from ASC was determined by the measurement of FGF‐1 and VEGF, in the medium collected from the cells, using the Magnetic Luminex Human Premixed Multi‐Analyte Kit. The concentration of growth factors was 21.7 ± 0.7 pg/mL for FGF‐1 and 95.6 ± 3.1 pg/mL for VEGF. DMEM only showed growth factor concentrations close to zero (Figure 1).

### 3.2 HUVEC undergoing EndMT present conformational changes

**Figure 2:**
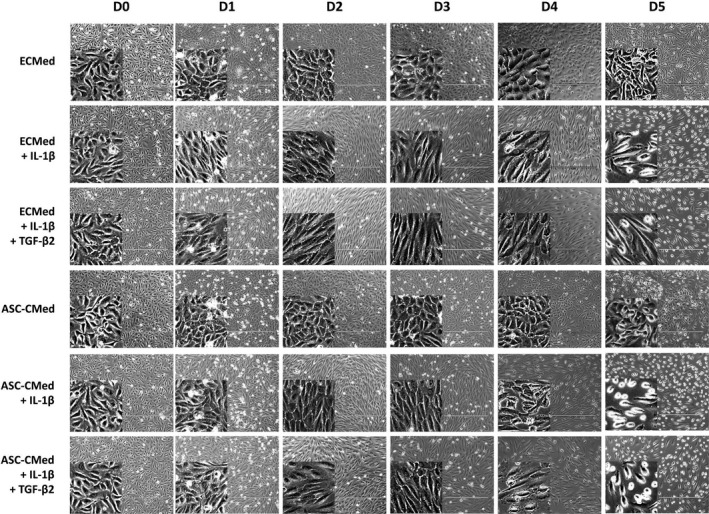
Conformational changes in HUVEC under stimulation with IL‐1β or co‐stimulation with IL‐1β/TGF‐β2, both in ECMed and in ASC‐CMed, for five days. Scale reference: 400 μm

All cells started the experiment as a cobblestone morphology (Figure 2). After two days, the cells receiving inflammatory stimuli had disrupted intercellular adhesions. During this same period, co‐stimulation with pro‐inflammatory and pro‐fibrotic factors, that is, induction of EndMT, part of the HUVEC showed more pronounced disruption of intercellular adhesions and had altered from their characteristic cobblestone morphology into spindle‐shaped cells (Figure 2). The cells cultured with ASC‐CMed retained their cobblestone morphology, but it did not inhibit the disruption of intercellular adhesions, in the cells neither with only inflammatory stimulation nor with both inflammatory and pro‐fibrotic stimulation (Figure 2). Control cells kept their morphology for the entire duration of the experiment. The inflammatory environment did not change the cells compared to the second day. In EndMT‐induced HUVEC, all intercellular adhesions were disrupted, while all cells were spindle‐shaped at day 5 (Figure 2). Although these changes could be seen both in the groups cultured only with IL‐1β and those undergoing co‐stimulation with IL‐1β and TGF‐β2, the latter showed a more explicit transformation. The use of ASC‐CMed did not prevent cell‐to‐cell adhesion disruption or morphology changes to occur.

### 3.3 Inflammatory gene expression in activated HUVEC is refractory to ASC‐secreted factors

**Figure 3:**
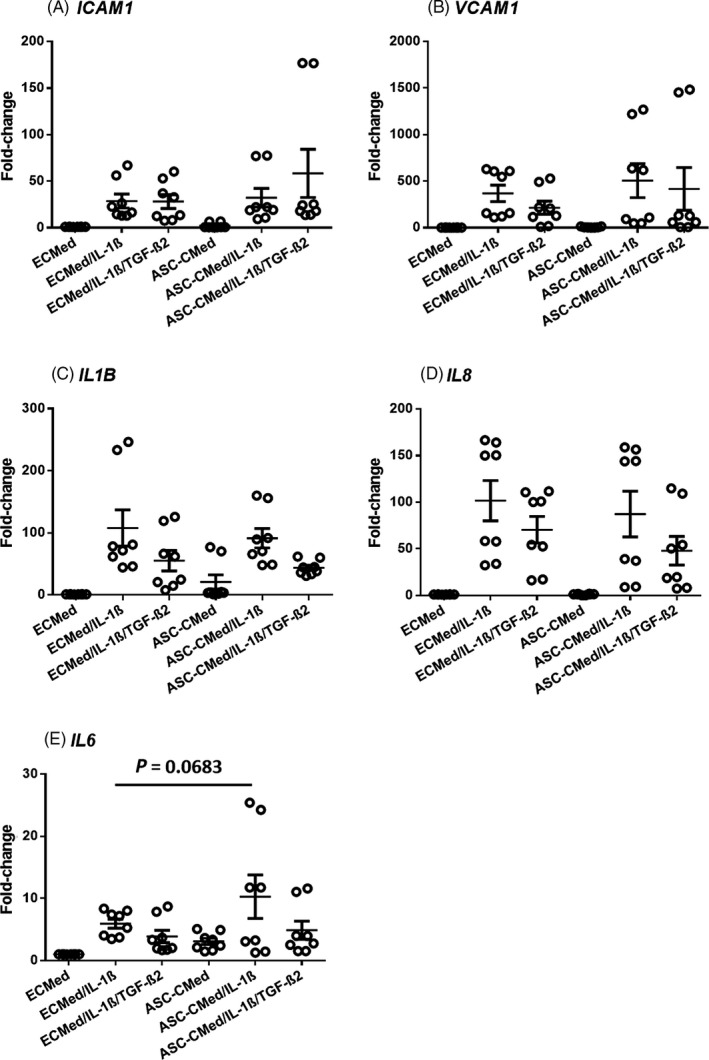
Gene expression (mRNA) of inflammatory markers A, *ICAM1*, B, *VCAM1*, C,* IL1B*, D, *IL8*, and E, *IL6* by semi‐quantitative RT‐qPCR of HUVEC after stimulation with IL‐1β or co‐stimulation with IL‐1β/TGF‐β2, both in ECMed and in ASC‐CMed, for five days. Data were analysed by one‐way ANOVA with Sidak's multiple comparison test for the groups ECMed/IL‐1β vs ASC‐CMed/IL‐1β; *P*‐values for the Sidak's multiple comparison test are shown in the figure. Values represent mean ± SEM of 4 independent experiments in duplicate

Pro‐inflammatory stimulation of HUVEC with IL‐1β upregulated expression of *IL8, ICAM1* and *VCAM1*, which encode respectively chemoattractant and adhesion molecules required for endothelial transmigration of activated leucocytes (Figure 3). This upregulation was refractory to simultaneous treatment with ASC‐CMed (Figure 3). Also, IL‐1β stimulation upregulated expression of two pro‐inflammatory cytokine genes, *IL1B* and *IL6*, which was unaffected by ASC‐CMed, except for *IL6* that was slightly upregulated by ASC‐CMed (one‐way ANOVA, *P* = 0.0075; Sidak's multiple comparison test, *P* = 0.0683). The influence of TGF‐β2 on IL‐1β–stimulated HUVEC was negligible with respect to the expression of these inflammatory activation‐related genes, neither did co‐stimulation with ASC‐CMed affect these genes. However, the expression of *IL6* was normalized compared to stimulation of HUVEC with IL‐1ß and ASC‐CMed.

### 3.4 Mesenchymal gene expression in EndMT‐induced HUVEC is suppressed by ASC‐secreted factors while extracellular matrix genes are not

**Figure 4:**
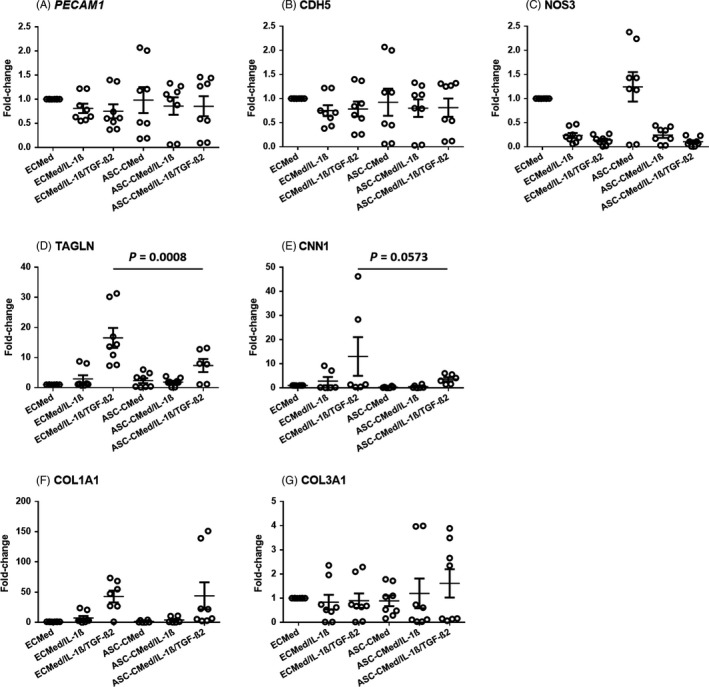
Gene expression (mRNA) of endothelial markers A, *PECAM1*, B, *CDH5* and C, *NOS3*; mesenchymal markers D, *TAGLN* and E, *CNN1;* and collagens F, *COL1A1* and G, *COL3A1* by semi‐quantitative RT‐qPCR of HUVEC under stimulation with IL‐1β or co‐stimulation with IL‐1β/TGF‐β2, both in ECMed and in ASC‐CMed, for five days. Data were analysed by one‐way ANOVA with Sidak's multiple comparison test for the groups ECMed/IL‐1β/TGF‐β2 vs ASC‐CMed/IL‐1β/TGF‐β2; *P*‐values for the Sidak's multiple comparison test are shown in the figure. Values represent mean ± SEM of four independent experiments in duplicate

Pro‐inflammatory stimulation of HUVEC with IL‐1β did not change the expression of *PECAM1* and *CDH5* (Figure 4A‐B) which are endothelial intercellular adhesion molecules that support the maintenance of the endothelial barrier. As expected, this pro‐inflammatory activation abolished eNOS (*NOS3)* gene expression (Figure 4C). Similarly, endothelial co‐stimulation with IL‐1β and TGF‐β2 did not affect the expression of *PECAM1* and *CDH5*, while *NOS3* expression was abolished too (Figure 4A‐C). Though TGF‐β2 has anti‐inflammatory effects, it could not alleviate the strong influence of IL‐1β on *NOS3* downregulation. The expression of these endothelial‐specific genes was unaffected by ASC‐CMed neither in unstimulated controls nor after cytokine activation (Figure 4A‐C). The expression of *TAGLN* and *CCN1*, mesenchymal genes typical for EndMT, was unaffected in HUVEC after pro‐inflammatory stimulation (Figure 4D‐E). As expected, co‐stimulation with IL‐1ß and TGF‐ß2 upregulated expression of *TAGLN* and* CCN1*. Endothelial gene expression (*PECAM1*, *CDH5,* and *NOS3*) was not affected by ASC‐CMed in controls or cytokine‐stimulated HUVEC. On the other hand, ASC‐CMed normalized expression of *TAGLN* (Figure 4D, one‐way ANOVA, *P* < 0.0001; Sidak's multiple comparison test, *P* = 0.0008) and, albeit to a lesser extent, of *CCN1* (Figure 4E, one‐way ANOVA, *P* = 0.0976; Sidak's multiple comparison test, *P* = 0.0573) in HUVEC that were induced to undergo EndMT, that is, co‐stimulation with IL‐1β and TGF‐β2 (Figure 4D‐E). Over the five‐day period of the pro‐inflammatory induction or the induction of EndMT, the expression of representative fibrosis‐related extracellular matrix genes *COL1A1* and *COL3A1* was upregulated after co‐stimulation with both cytokines (Figure 4F‐G). In contrast to the structural mesenchymal genes *TAGLN* and *CCN1*, the upregulation of *COL1A1* and *COL3A1* was refractory to treatment with ASC‐CMed (Figure 4F‐G).

**Figure 5:**
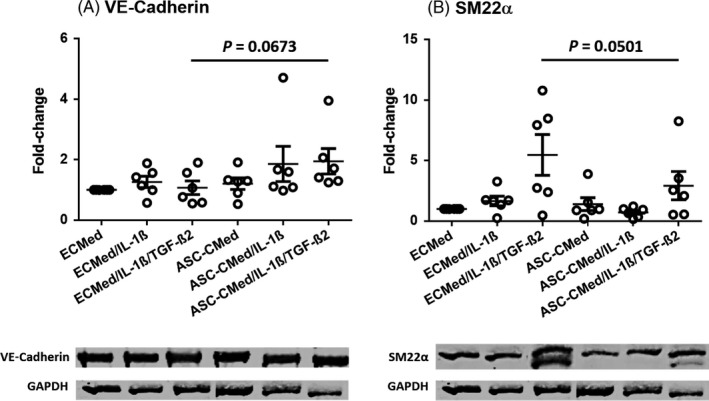
Protein expression of A, VE‐cadherin and B, SM22ɑ by Western blot of HUVEC under stimulation with IL‐1β or co‐stimulation with IL‐1β/TGF‐β2, both in ECMed and in ASC‐CMed, for five days. Data were analysed by one‐way ANOVA with Sidak's multiple comparison test for the groups ECMed/IL‐1β/TGF‐β2 vs ASC‐CMed/IL‐1β/TGF‐β2; *P*‐values for the Sidak's multiple comparison test are shown in the figure. Values represent mean ± SEM of 6 independent experiments

To corroborate the gene expression results, protein expression was assessed by immunoblotting of the endothelial marker VE‐cadherin (CD144, *CDH5* gene) and the mesenchymal marker SM22 (transgelin, *TAGLN* gene) (Figure 5). The expression of both proteins did not change in upon pro‐inflammatory stimulation, nor was it affected by co‐treatment with ASC‐CMed. The expression of VE‐cadherin, however, was slightly increased by ASC‐CMed, irrespective of cytokine treatment (Figure 5A, one‐way ANOVA, *P* = 0.1990; Sidak's multiple comparison test, *P* = 0.0673). The upregulated expression of SM22 after stimulation with both cytokines was suppressed by ASC‐CMed (Figure 5B, one‐way ANOVA, *P* = 0.0064; Sidak's multiple comparison test, *P* = 0.0501).

**Figure 6:**
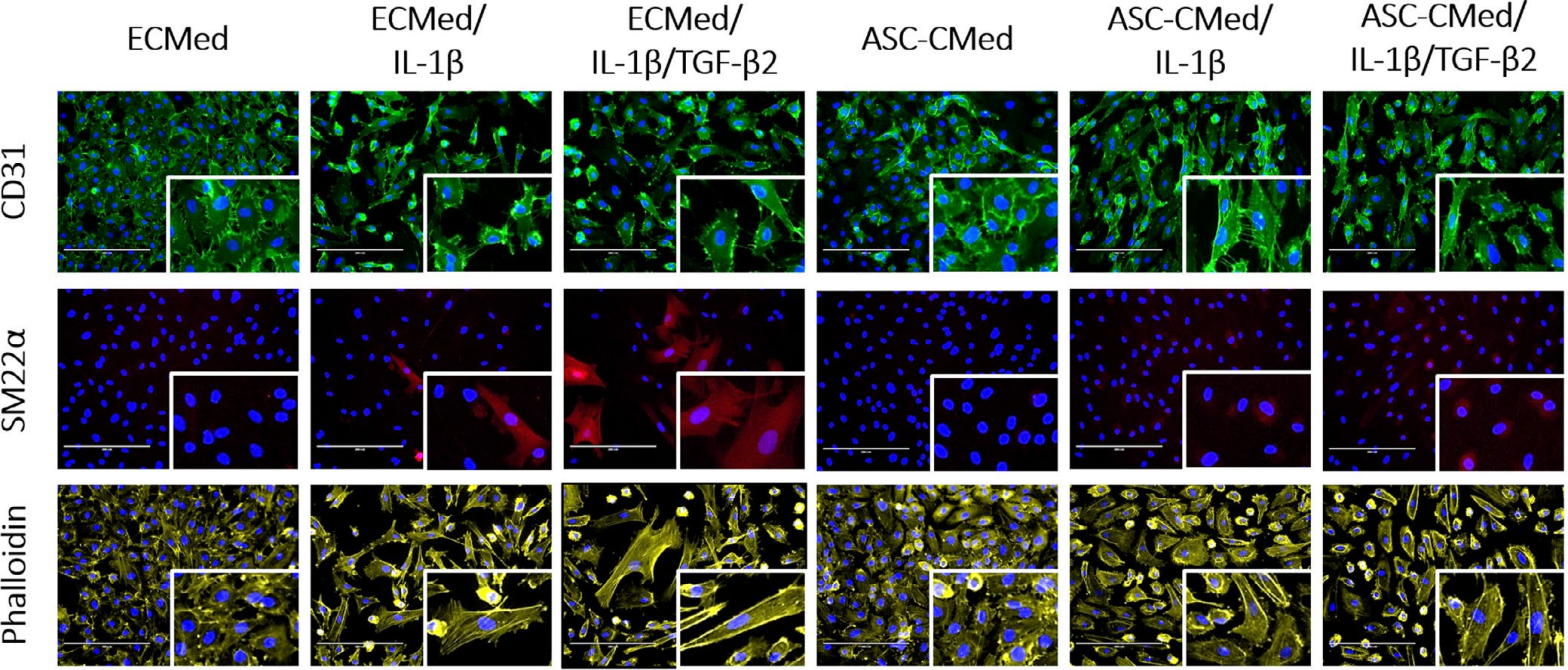
Fluorescence microscopy for phalloidin, SM22ɑ, and CD31 of HUVEC after stimulation with IL‐1β or co‐stimulation with IL‐1β/TGF‐β2, both in ECMed and in ASC‐CMed, for five days. Scale reference: 100 μm

Immunofluorescence staining for the endothelial marker PECAM (CD31) showed that none of the cytokine treatments, nor the co‐treatment with ASC‐CMed, affected its expression (Figure 6, top row panels). In contrast to immunoblotting, in situ immunostaining of SM22α proved less sensitive, yet it was detectable after stimulation with IL‐1β alone or together with TGF‐β2. Upon co‐treatment with ASC‐CMed, SM22α expression was below detectable levels, irrespective of treatment (Figure 6, middle row panels). This indicates that ASC secrete factors that suppress SM22α in cytokine‐stimulated HUVEC. The five‐day pro‐inflammatory activation of HUVEC induced hypertrophy as judged by F‐actin detection with phalloidin staining (Figure 6, the lower row of panels). The hypertrophy was stronger and associated with transcellular stress fibres in HUVEC induced to undergo EndMT. Co‐treatment with ASC‐CMed, at least qualitatively, reduced the hypertrophy and intracellular stress fibres in HUVEC induced that underwent EndMT.

Downstream TGF‐ß signalling in EndMT is governed by the complex of the canonical TGF‐β type II receptor (*TGFBR2*) and the TGF‐β type I receptor ALK5 (*ALK5*) that activate any of the transcription factors Snail (*SNAI1*), Slug (*SNAI2*) or Twist (*TWIST1*). The expression of *TGFBR2* remained unchanged, irrespective of cytokine treatment or co‐treatment with ASC‐CMed (Figure S1 A). However, as we published before, *ALK5* expression increased upon stimulation of EndMT for five days, albeit not significantly (Figure S1 B). The co‐treatment with ASC‐CMed did not influence the expression of *ALK5* irrespective of cytokine treatment. Expression of the most relevant downstream EndMT‐associated transcription factor *SNAI1* paralleled the expression pattern of *ALK5,* that is, upregulation by co‐stimulation with IL‐1β and TGF‐β2, while ASC‐CMed had no influence on its expression (Figure S1 C). The expression of the second relevant transcription factor *SNAI2* was unaffected except for treatment with both cytokines and the ASC‐CMed (Figure S1 D, one‐way ANOVA, *P* = 0.0463; Sidak's multiple comparison test, *P* = 0.0244). Expression of *TWIST1* was unchanged but tended to be upregulated in the presence of ASC‐CMed (Figure S1 E).

### 3.5 Factors secreted by ASC fail to restore impaired sprouting capacity of HUVEC undergoing EndMT

**Figure 7:**
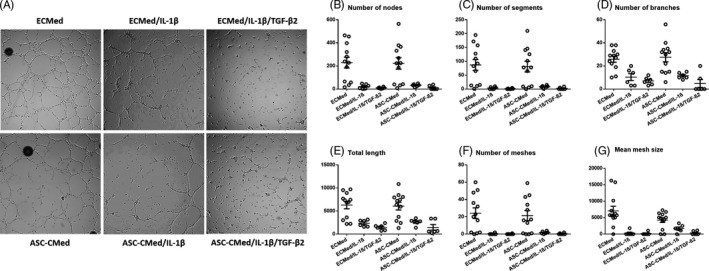
A, Sprouting assay (8 h) of HUVEC under stimulation with IL‐1β or co‐stimulation with IL‐1β/TGF‐β2, both in ECMed and in ASC‐CMed, for five days. Brightfield microscopy, augmentation 2.5X. Quantification of B, number of nodes, C, number of segments, D, number of branches, E, number of meshes, F, total length and G, mean mesh size. Values represent mean ± SEM of three independent experiments in duplicate

Endothelial cell function was assessed by short‐term sprouting on Matrigel® and quantified through determination of nodes, segments, branches, total length, meshes and mean mesh size (Figure 7). Pro‐inflammatory activated (5d, IL‐1ß) HUVEC or HUVEC undergoing EndMT (5d, IL‐1ß/TGF‐ß2), largely lost their sprouting capacity, although this was more explicit in the latter group (Figure 7). Treatment with ASC‐CMed did not restore the sprouting capacity of HUVEC, while control treatment of HUVEC with ASC‐CMed did not influence sprouting.

## 4 DISCUSSION

The aim of our investigation was to assess the impact of factors secreted by ASC on EndMT induced by co‐stimulation with IL‐1β and TGF‐β2. The main result is that ASC‐CMed normalized or even promoted endothelial markers after EndMT induction, while constructive mesenchymal markers were suppressed. However, these ASC‐secreted factors could not rescue the compromised endothelial functional phenotype because EC remained pro‐inflammatory activated and had severely blunted sprouting capacity. Thus, the treatment of pro‐inflammatory and pro‐fibrotic‐induced EndMT in vivo, such as during cardiac fibrosis, likely does not prevent endothelial dysfunction but might delay or suppress the mesenchymal transition itself, while more distant from a lesion ASC may still augment vascularization.

EndMT has been shown as an important process for the generation of myofibroblasts and, thus, fibrosis.^3^ The potential of ASC to inhibit EndMT may be one of the mechanisms involved in myocardial regeneration following cell therapies based on ASC.^34^, ^35^, ^36^, ^37^, ^38^, ^41^ Literature supports that growth factors known to be secreted by ASC—such as FGF and VEGF 53, 54—could block EndMT.^55^, ^56^, ^57^ Besides growth factors, the ASC secretome comprises microRNAs (often secluded in exosomes), among which are miR‐155, miR‐31, and miR‐21, all known regulators of EndMT.^16^, ^58^, ^59^ Another mechanism that may influence the expression of SM22ɑ is an epigenetic modification, for instance, the trimethylation of histone three (H3K27me3) by enhancer of zeste homolog 2 (EZH2)^30^

Previously, mesenchymal cells derived from menstrual blood (MMC) were shown to ameliorate cardiac fibrosis via inhibition of EndMT in myocardial infarction.^60^ The authors showed that the total number of cells co‐expressing CD31 and ɑSMA in the infarcted heart was reduced from 30% in the control group to 20% in the group treated with MMC. In our in vitro study, we also showed that the inhibition of EndMT occurred in a limited manner, corroborating the findings of the in vivo study, which showed the complete blockage of the EndMT process could not be achieved in vivo. The percentage of cells co‐expressing endothelial and mesenchymal markers in the sham group, as a reference, was less than 4% (compared to the 20% in the group treated with MMC), but even with the moderate reduction evidenced in the treated group, modulation of cardiac damage could be demonstrated by the reduction in the infarcted area. The use of ASC, in turn, was demonstrated to inhibit epithelial‐to‐mesenchymal transition (EMT) and consequently renal fibrosis.^61^, ^62^ Analogously to EndMT, EMT is a fibrotic process induced by TGF‐β and mediated by key transcription factors such as Smad2/3, Snail and Twist.^63^, ^64^ The effects demonstrated with the use of ASC in EMT are an important indicator that these cells would also play a role in EndMT; thus, our findings on endothelial cells are also in agreement with the findings described for epithelial cells.

The detailed underlying molecular mechanism of EndMT blockage was not dissected in the present study. We expected a decrease in *SNAI1*, *SNAI2* and *TWIST1* expression after use of ASC‐CMed because these are transcription factors involved in TGF‐β–induced EndMT.^25^, ^65^ In contrast, we found that *SNAI2* was overexpressed when HUVEC co‐stimulated with IL‐1β/TGF‐β2 were cultured in ASC‐CMed, while no differences were found for *SNAI1* or *TWIST1*. Still, it was described in the literature that although EndMT is associated with an increased expression of *SNAI2,* the overexpression of *SNAI2* alone is not enough to promote EndMT, being also required the inhibition of the *SNAI2* inhibitor GSK‐3β.^23^ The GSK‐3β, in turn, is inhibited by Smad2/3,^66^ which is recruited by TGF‐β.^67^ Thus, in the hypothesis that ASC‐CMed would interrupt the canonical TGF‐β pathway, Smad2/3 would be decreased and GSK‐3β would not be inhibited, consequently blocking the SNAI2. Still, besides the predominant TGF‐β canonical pathway, the non‐canonical pathway was also described as mediating EndMT.^68^, ^69^ Other mechanisms involve the AKT signalling pathway, via the FOXO3 transcription factor,^70^, ^71^, ^72^ and the MAPK/ERK pathway, via the ELK1 transcription factor.^28^ Besides these pathways, the study of exosomes and miRNAs has emerged in the past few years, showing the presence of several entities involved in the EndMT process, such as mi21, mi146, let7 12, 72, 73

## 5 CONCLUSION

The present study supports the anti‐fibrotic effects of ASC‐CMed through the modulation of the endothelial‐mesenchymal transition process. We demonstrated that ASC‐CMed reduces EndMT induced by co‐stimulation with IL‐1β and TGF‐β2 as evidenced by the reduction in expression of mesenchymal markers. Still, further investigations are needed to elucidate the exact underlying mechanisms.

## CONFLICT OF INTEREST

The authors declare no conflicts of interest.

## AUTHOR CONTRIBUTION

TTAL has contributed to the conception and design of the work, data collection, data analysis and interpretation, drafting the article, critical revision of the article and final approval of the manuscript text. GRL has contributed to the conception and design of the work, data collection, data analysis and interpretation, drafting the article, critical revision of the article and final approval of the manuscript text. LFPM has contributed to the data analysis and interpretation, critical revision of the article and final approval of the manuscript text. MCH has contributed to the conception and design of the work, data analysis and interpretation, critical revision of the article and final approval of the manuscript text.
