# Supplementary material for: Adipose tissue–derived stromal cells’ conditioned medium modulates endothelial‐mesenchymal transition induced by IL‐1β/TGF‐β2 but does not restore endothelial function
Source: Cell Prolif. 2019 Aug 29;52(6):e12629. doi: 10.1111/cpr.12629 (PMC6869467; doi:10.1111/cpr.12629)
Supplement: Supplementary file 1 [file CPR-52-e12629-s001.docx]

**SUPPLEMENTARY MATERIAL**

| **Table S1. Primer Sequences for RT-qPCR** | | | |
| --- | --- | --- | --- |
|  | **Gene** | **Forward Primer Sequence (5’ – 3’)** | **Reverse Primer Sequence (5’ – 3’)** |
| **Reference Gene** | ***RNA18S*** | GCAATTATTCCCCATGAACG | GGGACTTAATCAACGCAAGC |
| **Inflammation Markers** | ***VCAM1*** | TGGACATAAGAAACTGGAAAAGG | CCACTCATCTCGATTTCTGGA |
|  | ***ICAM1*** | CCTTCCTCACCGTGTACTGG | AGCGTAGGGTAAGGTTCTTGC |
|  | ***IL1B*** | AAGCTGGAATTTGAGTCTGC | ACACAAATTGCATGGTGAAG |
|  | ***IL6*** | AGCTCAATAAGAAGGGGCCTA | TGAGAAACCCTGGCTTAAGTAGA |
|  | ***IL8*** | CTTTCAGAGACAGCAGAGCA | ACACAGAGCTGCAGAAATCA |
| **Endothelial Markers** | ***PECAM 1*** | GCAACACAGTCCAGATAGTCGT | GACCTCAAACTGGGCATCAT |
|  | ***CDH5*** | GTTCACCTTCTGCGAGGATA | GTAGCTGGTGGTGTCCATCT |
|  | ***NOS3*** | CACATGGCCTTGGACTGAA | CAGAGCCCTGGCCTTTTC |
| **Mesenchymal Markers** | ***TAGLN*** | CTGAGGACTATGGGGTCATC | TAGTGCCCATCATTCTTGGT |
|  | ***CNN1*** | CCAACCATACACAGGTGCAG | TCACCTTGTTTCCTTTCGTCTT |
| **ECM Markers** | ***COL1A1*** | GGGATTCCCTGGACCTAAAG | GGAACACCTCGCTCTCCA |
|  | ***COL3A1*** | CTGGACCCCAGGGTCTTC | CATCTGATCCAGGGTTTCCA |
| **Transcription Factors/Receptors** | ***SNAI1*** | GCTGCAGGACTCTAATCCAGA | ATCTCCGGAGGTGGGATG |
|  | ***SNAI2*** | TGGTTGCTTCAAGGACACAT | GTTGCAGTGAGGGCAAGAA |
|  | ***TWIST1*** | AAGGCATCACTATGGACTTTCTCT | GCCAGTTTGATCCCAGTATTTT |
|  | ***ALK5*** | AAATTGCTCGACGATGTTCC | CATAATAAGGCAGTTGGTAATCTTCA |
|  | ***TGFBR2*** | TCCATCTGTGAGAAGCCACA | GGGTCATGGCAAACTGTCTC |

**
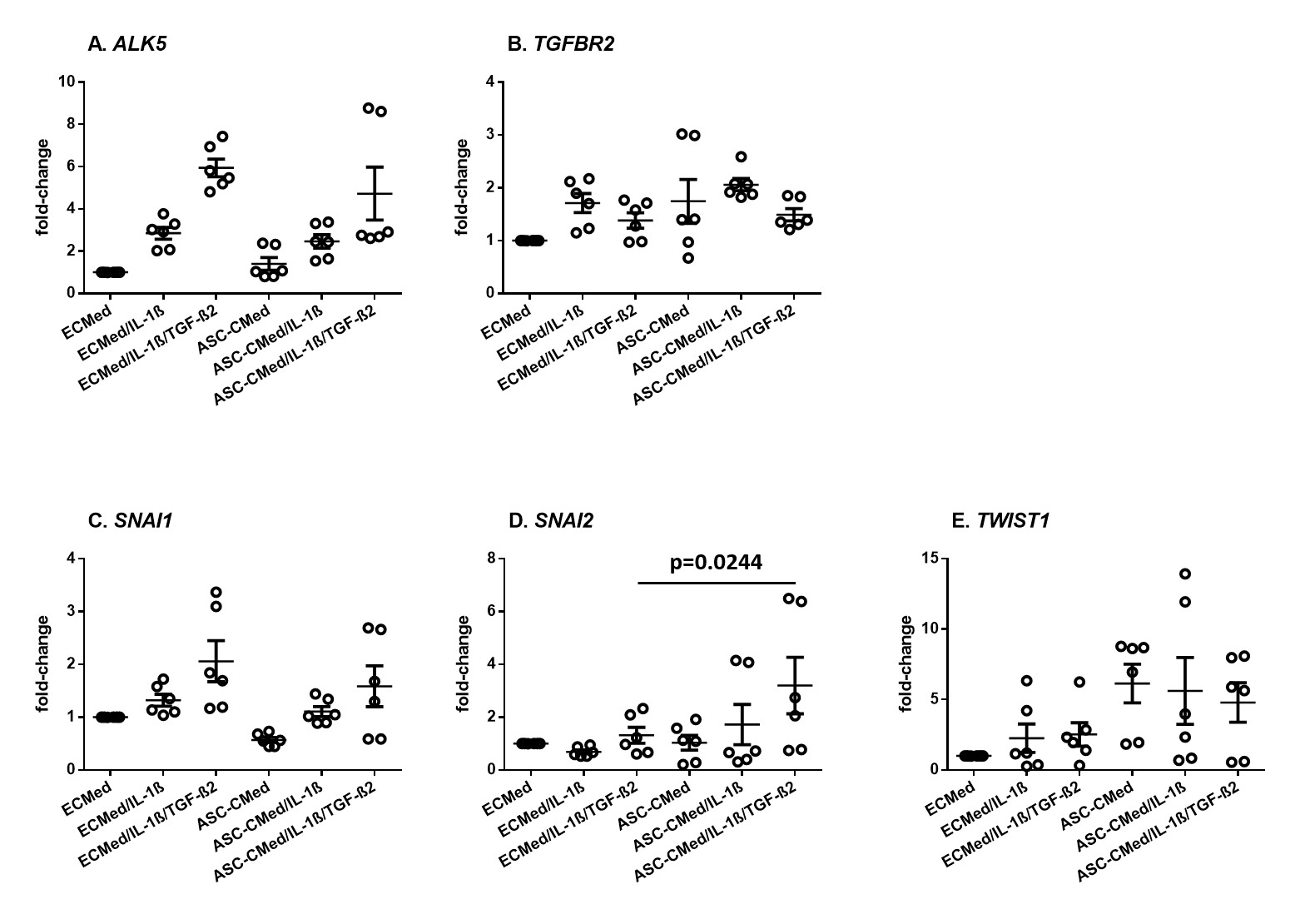
**

**Fig. S1** Gene expression (mRNA) of cell receptors A) *ALK5* and B) *TGFBR2,* and transcription factors C) *SNAI1*, D) *SNAI2*, and E) *TWIST1* by semi-quantitative RT-qPCR of HUVEC under stimulation with IL-1β or co-stimulation with IL-1β/TGF-β2, both in ECMed and ASC-CMed, for five days. Data were analyzed by One-way ANOVA with Sidak’s multiple comparison test for the groups ECMed/IL-1β/TGF-β2 vs. ASC-CMed/IL-1β/TGF-β2; p-values for the Sidak’s multiple comparison test are shown in the figure. Values represent mean ± SEM of 3 independent experiments in duplicate.
